# Analogies between the periphery of cancer and the leading edge of pulmonary fibrosis

**DOI:** 10.1186/s12967-023-04096-5

**Published:** 2023-04-21

**Authors:** Monica Pernia Marin, Mary Salvatore

**Affiliations:** Columbia University Irving Medical Center, New York, NY USA

## Abstract

The periphery of malignant tumors and the leading edge of fibrotic tissue have analogous metabolic pathways. Both use glycolysis as the primary source of energy to produce biomass with consequential acidification of the microenvironment. A low PH has been shown to increase the ability of cancer cells to invade the surrounding tissue in both in vitro and in vivo studies. The pH-dependent activation of TGF-B leading to myofibroblast activation is an important step in the initiation and progression of fibrosis. Markers of accelerated cell proliferation have also been reported in the periphery of malignant tumors and the leading edge of fibrosis. Understanding the shared molecular and metabolic characteristics of these conditions may explain the increased prevalence of cancer among patients with fibrosis.

Anaerobic glycolysis utilizes one glucose molecule and 2 ATP to create 4 ATP, 2 pyruvates, and 2 NADH [1] (Fig. 1). Pyruvate is converted to lactate in the cytoplasm and transported to the extracellular matrix in a hypoxic environment. In the presence of oxygen, pyruvate enters the mitochondria and joins with coenzyme A to form acetyl-CoA in the Krebs cycle and ultimately yields 2 ATP, 2 FADH2, and 6 NADH. Electron transport phosphorylation in the mitochondria converts 2 FADH2 and 6 NADH to 32 ATP [1]. Tumors preferentially use glycolysis for energy production despite the relatively low levels of ATP production in what is called the Warburg effect. The hypothesized reasons for a switch to glycolysis include abnormal tumor mitochondria preventing oxidative phosphorylation, relative hypoxia, the need for more rapid ATP production, and the creation of biomass which seems most likely [2]. The excess carbon produced by glycolysis in the form of lactate allows faster incorporation of carbon into biomass, which in turn facilitates rapid cell division beneficial for a growing tumor [3].

**Fig. 1:**
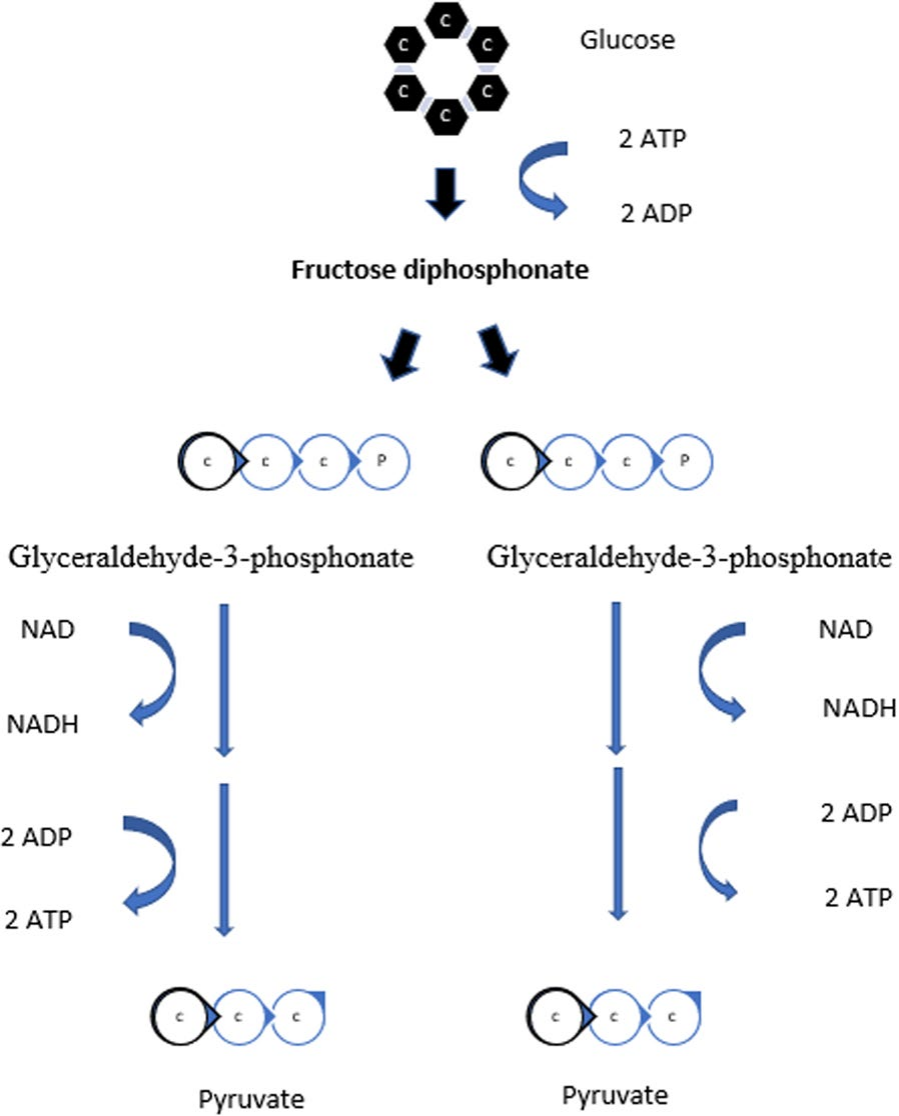
Diagrammatic representation of the production of 4 ATP, 2 pyruvates, and 2 NADH from one glucose molecule and 2 ATP through anaerobic glycolysis

Tumors are heterogeneous and their heterogeneity can be validated with radiologic imaging (Fig. 2). Gillies described how the core of a tumor is different from its edge and that the interface between the tumor and stroma provides prognostic value [4]. Lloyd et al. found that Ki67, a marker of cell proliferation, is much higher at the edge of a tumor which is not surprising given the tumor’s need to increase biomass at its *leading edge* [5]. The majority of cells in the human body use oxidative phosphorylation (OXPHOS) for ATP production as a source of energy, however, when cells are actively proliferating in regenerating tissues or cancer, they need to increase their biomass with the anabolic building blocks provided by glycolysis [6].

**Fig. 2:**
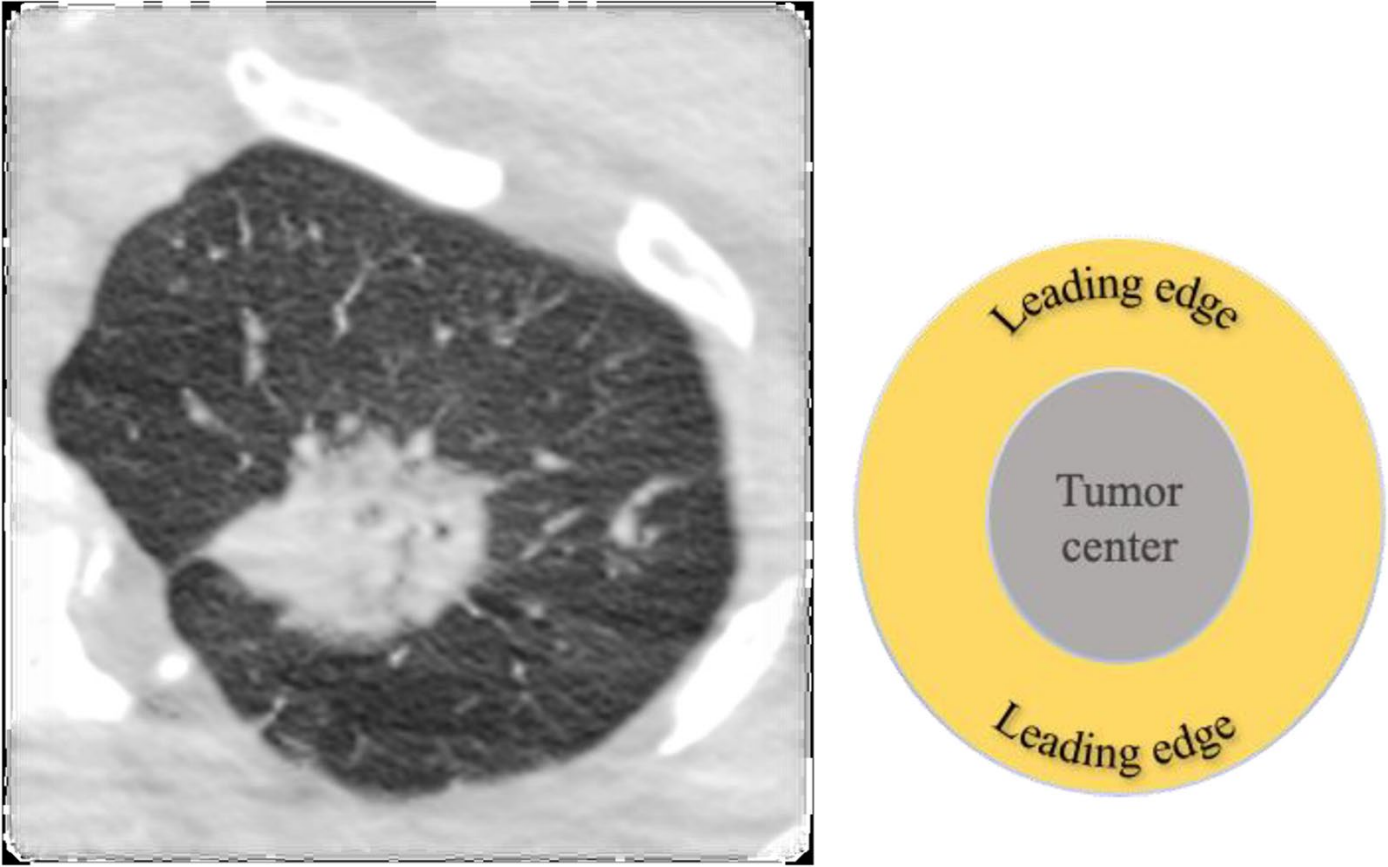
Heterogenous lung tumor showed on CT scan (photograph detail from an axial cut). On the right, a diagram illustrating the difference between the center and the leading edge of the tumor

In further support of increased glycolysis at the tumor periphery, Lloyd found increased Glut1 associated with the utilization of glucose. He identified increased carbonic anhydrase 9, a marker of extracellular acidity. Carbonic anhydrase 9 participates in the mobilization of hydrogen ions in the cytoplasm to the extracellular matrix [4, 5]. The hypoxia-inducible factor 1 (HIF1) is of paramount importance in regulating the body’s response to hypoxia at the cellular level. HIF is composed of 2 proteins; HIF-1B and HIF-1 alpha or HIF-2 alpha. HIF-1 alpha responds to low oxygen caused by increased biomass or decreased oxygen availability due to distance from vessels and facilitates the process of angiogenesis. HIF-1 alpha affects tumor metabolism including the utilization of glucose and the production of lactate [7].

We extrapolated these tumor metabolism principles to idiopathic pulmonary fibrosis (IPF) where the radiographic pattern is usual interstitial pneumonitis characterized as subpleural fibrosis with honeycombing (Fig. 3). Repeated micro-injury to the alveolar epithelial cells has been recognized as the initiating event in the altered repair process of IPF [8]. As pulmonary fibrosis progresses the *leading edge* of the fibrosis moves from peripheral to central in the lung. The laying down of collagen requires biomass production which would require a shift to glycolysis. Glycolysis increases lactate production causing a lowering of the PH in the periphery of the fibrosis where there is progression. Analogous to the periphery of a tumor, we would expect elevated HIF-1alpha due to the relative hypoxia and elevated Glut1 due to glucose utilization and elevated CA9 related to acidosis secondary to extra lactate. Zhao provided proof of these concepts by studying fibrotic lungs where he found no change in the early-stage glycolysis metabolites, glucose and fructose 6-phosphate, but significantly decreased levels of later-stage glycolysis metabolites compared with normal lungs. They also found increased lactic acid levels [9].

**Fig. 3:**
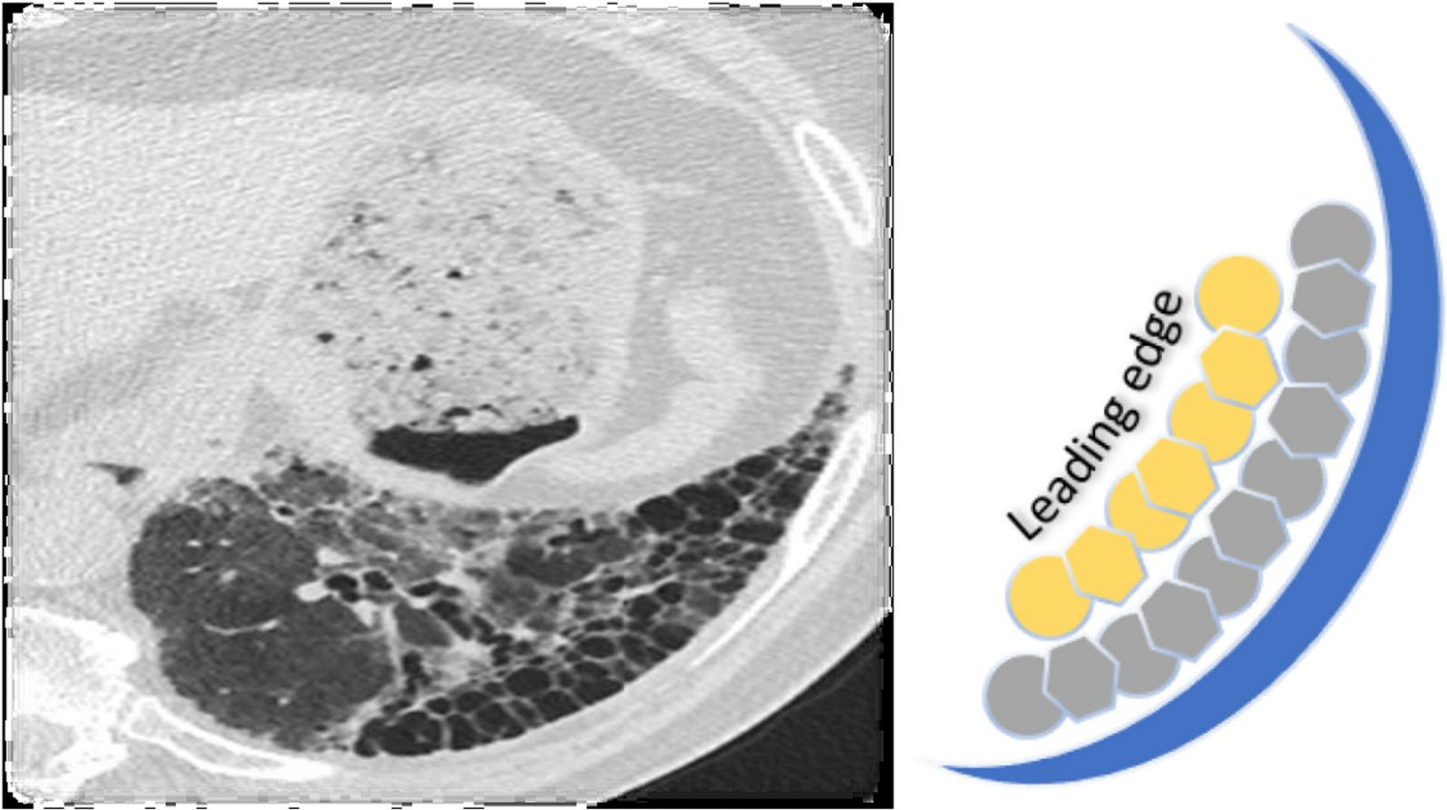
Idiopathic pulmonary fibrosis observed on CT scan (photograph detail from an axial cut). On the right, a diagram illustrating the presence of the leading edge of the fibrotic tissue replacing healthy lung parenchyma from the periphery to the center

Cancer is increased in patients with pulmonary fibrosis (Fig. 4). Kottmann et al. identified lactic acid as an important mediator of myofibroblast differentiation via a pH-dependent activation of TGF-β. It plays a potential role in the initiation and progression of fibrosis [10]. Epstein et al. provided further support that HIF-1 alpha promotes fibrosis [11]. Lactic acidosis can cause a phenotypic switch of macrophages from M1 to M2, which is the form involved in tissue remodeling [12].

**Fig. 4:**
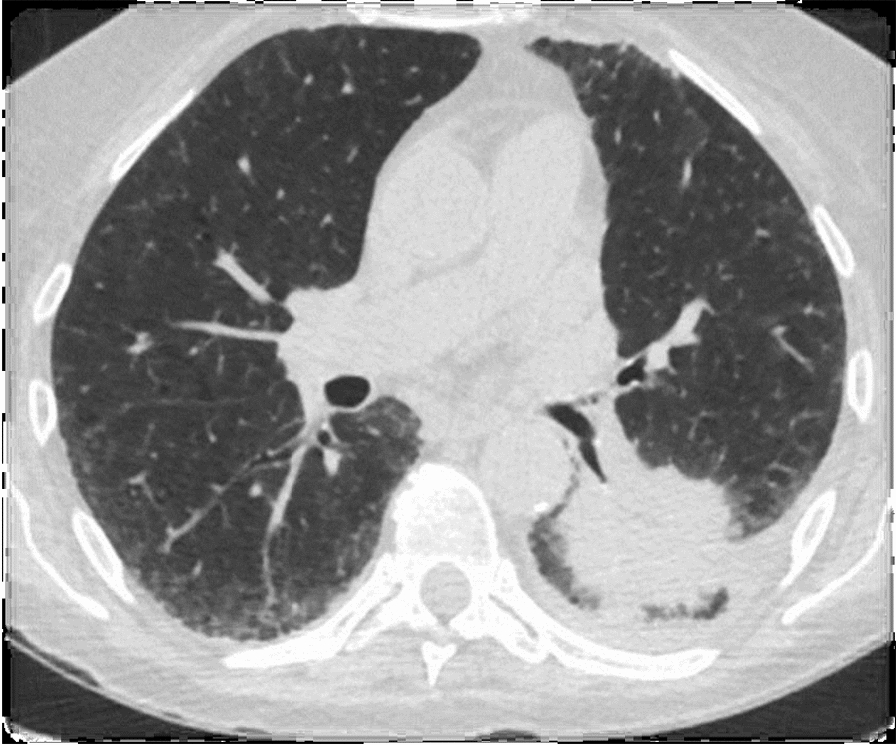
Malignant lung tumor in a patient with pulmonary fibrosis observed on CT scan

PET imaging with FDG demonstrates how both cancer cells and pulmonary fibrosis utilize glycolysis as an energy resource (Fig. 5) [13]. 18F-FDG PET/CT has been studied in pulmonary fibrosis. There is increased pulmonary 18F-FDG metabolism corresponding to fibrosis on CT. The authors found that the metabolism was greater in areas of reticulation compared with areas of ground glass [14].

**Fig. 5:**
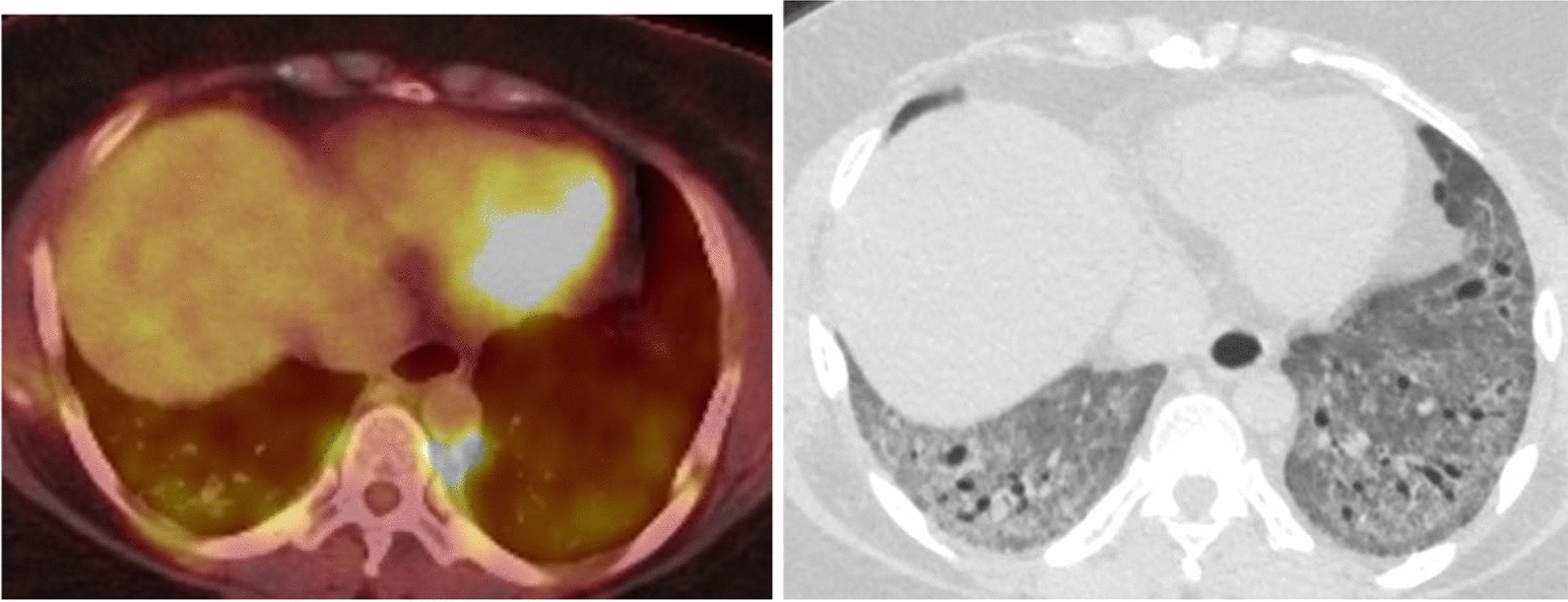
PET imaging with FDG demonstrates how both cancer cells and pulmonary fibrosis utilize glycolysis as an energy resource

## Conclusion

The need for increased biomass at the *leading edge* of tumors and fibrosis cause them both to rely on glycolysis with its resulting acidic microenvironment. This low pH microenvironment with its effect on RAS and TGF-B could explain at least in part the risk for cancer in fibrosis [15]. PET imaging with its ability to identify the highest areas of glucose utilization may provide deeper insight. Further investigation will determine if imaging supports this hypothesis.
